# Discovery of a novel tetrapeptide as glucose homeostasis modulator with bifunctionalities of targeting DPP‐IV and microbiota

**DOI:** 10.1002/imt2.70072

**Published:** 2025-08-11

**Authors:** Haihong Chen, Wei Li, Wei Hu, Junyu Liu, Canyang Zhang, Yi Wang, Chong Zhang, Xizhen Zhang, Shuo Chen, Qixing Nie, Xinhui Xing

**Affiliations:** ^1^ Institute of Biopharmaceutical and Health Engineering Tsinghua Shenzhen International Graduate School Shenzhen China; ^2^ Key Laboratory of Active Proteins and Peptides Green Biomanufacturing of Guangdong Higher Education Institutes Tsinghua Shenzhen International Graduate School Shenzhen China; ^3^ Key Laboratory for Industrial Biocatalysis, Ministry of Education, Institute of Biochemical Engineering Department of Chemical Engineering Beijing China; ^4^ Center for Synthetic and Systems Biology Tsinghua University Beijing China; ^5^ College of life Science and Technology Guangxi University Nanning China; ^6^ Department of Psychiatry New York University Grossman School of Medicine New York New York USA; ^7^ State Key Laboratory of Food Science and Resources, China‐Canada Joint Lab of Food Science and Technology, Key Laboratory of Bioactive Polysaccharides of Jiangxi Province Nanchang University Nanchang China

## Abstract

AI‐driven and computation‐based high‐throughput methods were developed to mine novel dipeptidyl peptidase IV (DPP‐IV) inhibitory peptides from hemp seed proteins. The identified peptide VAMP demonstrates a glucose‐lowering effect through dual mechanisms: inhibition of DPP‐IV activity and selective promotion of intestinal *Akkermansia muciniphila* growth.

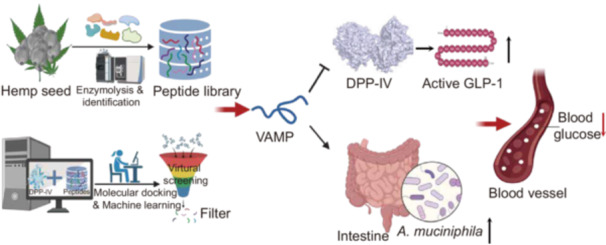

To the Editor,

Disordered glucose metabolism is closely linked with diabetes, obesity, liver disease, and several cardiovascular diseases [1]. Exploration of effective approaches to alleviate glucose metabolic disorders has gained increasing attention [2]. L cells derived glucagon‐like peptide‐1 (GLP‐1) has been identified to play an important role in glucose homeostasis via stimulating insulin secretion. However, GLP‐1 is rapidly degraded by the enzyme dipeptidyl peptidase IV (DPP‐IV) in the intestine [3].

Currently, one of the important strategies receiving increasing attention is to mine food‐derived bioactive peptides from the abundant natural food protein sources for developing DPP‐IV bioinhibitors [4]. However, the lack of genomic and proteomic information for many natural protein‐containing bioresources also hinders the efficient mining of new DPP‐IV inhibitory peptides. Specifically, the main approaches for the mining of DPP‐IV inhibitory peptides are labor‐, time‐, and cost‐intensive, including the complicated procedure such as enzymatic protein hydrolysis, separation, purification, identification, and bioactivity examination by synthesis of the target peptides [5]. Therefore, it is challenging to develop a high‐throughput integrated mining method which enables effective DPP‐IV inhibitory biopeptides to be effectively discovered from natural protein‐rich bioresources that can be used in functional food development and peptide medicine innovation.

Hemp (*Cannabis sativa* L.) is widely farmed and has long been approved for medicinal use in China. Hemp seeds are rich in proteins (20%−25%), which contain various essential amino acids in a desirable ratio [6]. Our previous study indicated that hemp seed protein (HSP) hydrolysates possessed favorable DPP‐IV inhibitory effects, but the peptide composition and detailed mechanism have not yet been explored [7, 8]. Moreover, extensive studies have reported that bioactive peptides can improve glucose metabolism by altering gut microbiota. Since the site of action of DPP‐IV‐inhibiting peptides is primarily the intestine, it is an interesting question if the HSP‐derived DPP‐IV‐inhibiting biopeptides can multifunctionally affect the gut microbiota in the regulation of glucose metabolism. Here, we developed an integrated mining method to mine novel DPP‐IV‐inhibitory bioactive peptides and investigated their role in the regulation of gut microbiota, offering new insight into biopeptides discovery and hyperglycemia intervention.

### Screening of DPP‐IV‐inhibiting peptides from HSP

Enzymatic HSP hydrolysates derived by different enzymes, combined with in vitro and in vivo functional validation, were utilized to screen the functional enzymatic products derived from the HSP (Figure 1A). According to our measurements, the hemp seeds used in this study contained approximately 26% protein (Figure 1B and Table S1), and 1184 proteins were identified from the HSP via proteomic analysis (Table S2). Based on the functional annotation, HSP‐derived peptides have favorable DPP‐IV inhibitory effects (44% of the total annotated HSP‐derived peptides), implying that HSP‐derived peptides could contribute to improved glucose metabolism (Figure 1C).

**FIGURE 1:**
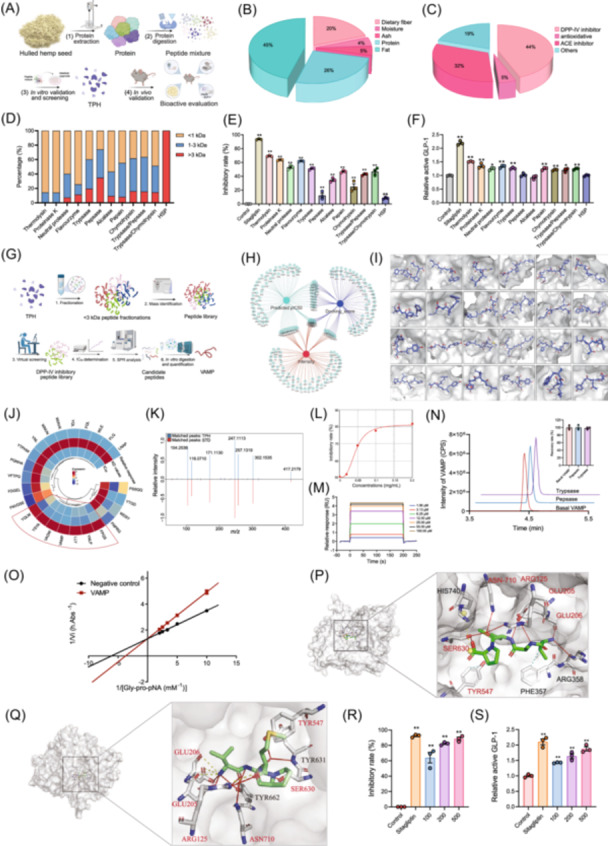
Mining of DPP‐IV‐inhibiting peptides in enzymatic products of HSP. (A) Strategy for screening enzymes to be used for the enzymolysis of HSP to obtain hydrolysates that inhibited DPP‐IV. (B) The nutritional composition of the hemp seeds was determined. (C) The percentage of peptide sequence numbers in HSP with various functions is shown. (D) The molecular weight distribution of the HSP hydrolysates generated using different enzymes. (E) The inhibitory rate of different HSP hydrolysates on DPP‐IV. (F) The relative concentrations of active GLP‐1 in intestinal organoid treated with different HSP hydrolysates. (G) Strategy for the identification of peptides that inhibit DPP‐IV. (H) Dynamic network Venn diagram analysis was performed for the top 100 peptides (tripeptides, tetrapeptides, pentapeptides, and hexapeptides) in TPH based on the intensity, affinity, and predicted pIC_50._ (I) The predicted binding model between DPP‐IV and the 24 selected bioactive peptides was visualized by PyMOL. (J) Heatmap analysis of the relative response, KD values, and IC_50_ values of the 24 selected peptides was performed. (K) The LC–MS data of VAMP from synthetic standards and TPH samples were compared. (L) The IC_50_ curve of VAMP. (M) The SPR sensorgrams of click‐immobilized DPP‐IV toward VAMP at different concentrations are shown. (N) The recovery rate of VAMP after pepsase and trypsase digestion was determined. (O) Lineweaver‒Burk plots of DPP‐IV inhibition by VAMP are shown. (P) Molecular docking results visualization of active‐site superimposition between VAMP‐bound DPP‐IV with key residues are labeled as a three‐letter code. VAMP (Green) and residues (White) are shown as sticks, and DPP‐IV is shown as surface. Hydrogen bonds are colored red, hydrophobic interactions are colored cyan, and salt bridges are colored yellow. (Q) X‐ray co‐crystal structures visualization of active site superimposition between VAMP‐bound DPP‐IV and key residues is labeled with a three‐letter code. VAMP (green) and residues (white) are shown as sticks, and DPP‐IV is shown as a surface. Hydrogen bonds are colored red and salt bridges are yellow. The red fonts represent the overlapping key residues for docking and X‐ray co‐crystal structure. (R) The inhibitory effect of VAMP on DPP‐IV was determined. (S) The relative concentrations of active GLP‐1 after VAMP treatment in intestinal organoids were measured. DPP‐IV, dipeptidyl peptidase IV; GLP‐1, glucagon‐like peptide‐1; HSP, hemp seed proteins; IC_50_, half maximal inhibitory concentration; KD, dissociation constant; LC–MS, liquid chromatography‐mass spectrometry; pIC_50_, predict half maximal inhibitory concentration; SPR, surface plasmon resonance; TPH, thermolysin‐catalyzed protein hydrolysates.

To investigate the DPP‐IV inhibitory spectrum of HSP‐derived peptides, 11 proteases that have been widely used in the food industry were applied for the enzymolysis of HSP (Figure 1D). Importantly, thermolysin‐catalyzed protein hydrolysates (TPH) possessed the strongest DPP‐IV inhibitory activity and increased the levels of active GLP‐1 in the organoid to the greatest extent (Figure 1E,F and Figure S1A,B). We next investigated the hypoglycaemic activity of TPH in vivo (Figure S1C). Supplementation with TPH ameliorated obesity‐related parameters, increased the total and active GLP‐1 in plasma, improved glucose tolerance, and reduced insulin resistance, suggesting TPH could improve glucose metabolism via inhibition of the DPP‐IV (Figure S1D−V).

### Mining of functional peptides with DPP‐IV inhibitory effects from TPH

The above results showed that TPH possessed strong DPP‐IV inhibitory activity and significantly improved glucose metabolism in vivo. Therefore, we explored the key active peptide sequences by combining molecular docking and machine learning‐based virtual screening, followed by in vitro functional evaluation of DPP‐IV inhibitory activity (Figure 1G). Based on the proteomics analysis, molecular docking (PyRx software), and neural network‐based virtual screening, 24 peptides were selected for further analysis (Figure 1H,I and Figure S2A−E). We also found the peptides VADW, VAMP, FNPRG, FLQ, WIAVK, YQLM, LLY, YSYA, FPQS, and WDSY showed relatively strong DPP‐IV inhibitory activities in vitro (Figure S2F). Specifically, seven peptides were found to have strong DPP‐IV inhibitory activity based on their affinity for DPP‐IV, half maximal inhibitory concentration (IC_50_) values, and relative surface plasmon resonance (SPR) response (Figure 1J), among which VAMP is the strongest DPP‐IV inhibitory peptide (Figure 1J−M and Figure S2G, Table S3). We next tested the digestive stability of these peptides, only VAMP, FPQS, and YNLP were readily resistant to the simulated gastric pepsase and trypsase digestion (Figure 1N and Figure S2H). Simultaneously, VAMP was the most abundant peptide in TPH (mean ± standard error: 4.58 ± 0.03 μg/mg; Figure S2I).

To investigate the mechanism by which VAMP inhibits DPP‐IV activity, we used Lineweaver–Burk double reciprocal representation methods to analyze the binding mode and showed that VAMP could competitively inhibit DPP‐IV activity (Figure 1O). We also co‐crystalized DPP‐IV with complex structure to a resolution of 1.80 Å, and VAMP could form hydrogen bonds with the DPP‐IV residues at ARG125, GLU205, GLU206, TYR547, SER630, TYR631, TYR662, and ASN710, and form salt bridges with the DPP‐IV residues at ARG125, GLU205, and GLU206, highly in consist with the molecular docking visualization result (Figure 1P,Q and Table S4). Furthermore, VAMP inhibited DPP‐IV and increased the level of active GLP‐1 in a dose‐dependent manner (Figure 1R,S).

### Systematic regulation of glucose metabolism by VAMP via intestinal DPP‐IV inhibition

As VAMP had the highest abundance in TPH and exhibited the strongest inhibitory activity against DPP‐IV, we treated high fat diet‐fed mice with VAMP for 1 week to examine the effects of VAMP on glucose homeostasis through the inhibition of intestinal DPP‐IV activity (Figure S3A). There were no significant differences in body weight or energy intake between the vehicle and VAMP groups (Figure S3B,C). However, VAMP treatment significantly decreased the intestinal DPP‐IV activity and increased the levels of intestinal and plasma active GLP‐1 without altering the intestinal and plasma total GLP‐1 levels (Figure S3D−H), which was associated with increased levels of glucose‐stimulated insulin and a better oral glucose tolerance test (Figure S3I−K).

We next assessed the effects of VAMP on model mice during the induction of abnormal glucose metabolism. VAMP treatment increased the levels of intestinal and plasma active GLP‐1, and improved insulin resistance and glucose tolerance (Figure 2A−H). Regulation of intestinal barrier function is important for ameliorating insulin resistance. We found treatment with VAMP significantly improved intestinal barrier function and reduced metabolic endotoxemia (Figure 2I−O). Furthermore, VAMP also improved glucose metabolism in *ob/ob* mice via inhibition of intestinal DPP‐IV activity (Figure S3L−W). Collectively, these results indicated that VAMP could systematically ameliorate glucose intolerance via the inhibition of intestinal DPP‐IV and improve intestinal barrier function in obese mice.

**FIGURE 2:**
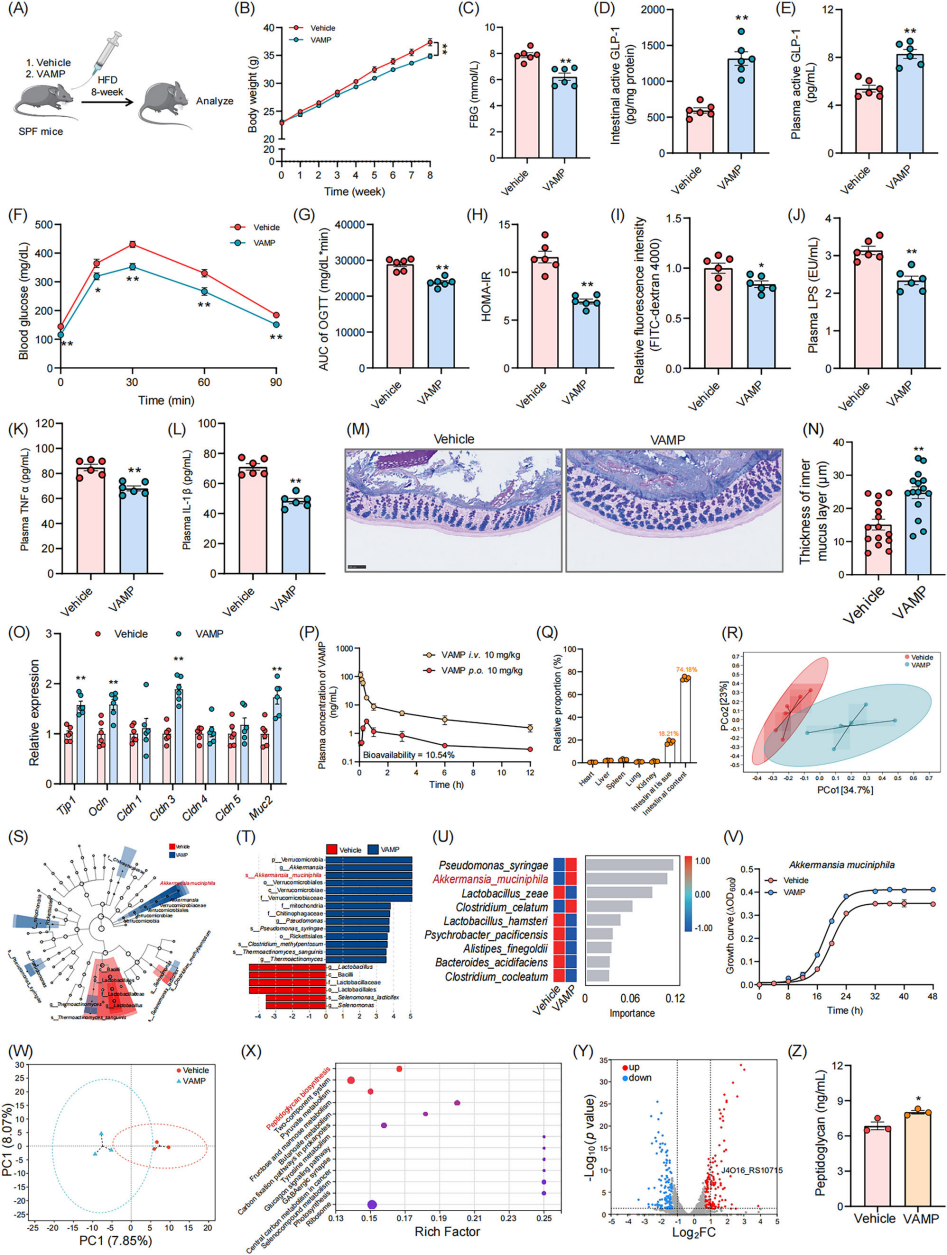
VAMP inhibits intestinal dipeptidyl peptidase IV (DPP‐IV) and increases the abundance of *A. muciniphila.* (A) The experimental scheme for (B–O) is shown, *n* = 6 mice/group. HFD‐fed mice were treated with PBS (vehicle group) or VAMP (50 mg/kg, VAMP group) for 8 weeks by oral gavage. (B) The body weight change, (C) FBG level, (D) intestinal active GLP‐1 levels, (E) plasma active GLP‐1 levels, (F) OGTT curve, (G) AUC of the OGTT, and (H) HOMA‐IR index were evaluated. (I) Intestinal permeability was measured by plasma fluorescence intensity after gavage with FITC‐dextran 4000. (J) The plasma levels of LPS, (K) TNF‐α, and (L) IL‐1β were determined. (M) Carnoy‐fixed colonic tissue sections were stained with Alcian blue/periodic acid‐Schiff. Scale bars, 100 µm. (N) Blinded colonic mucus layer measurements were made from Alcian blue‐stained sections. (O) The relative expression of *Tjp1*, *Ocln*, *Cldn1*, *Cldn3*, *Cldn4*, *Cldn5*, and *Muc2* mRNAs in colonic tissue was determined. (P) The blood pharmacokinetics of 10 mg/kg VAMP administered by injection (*i.v*.) or gavage (*p.o*.) over time were evaluated. (Q) The tissue distribution of VAMP at the time of peak plasma concentration (30 min) after the administration of 10 mg/kg VAMP by gavage was determined (*n* = 4). (R) PCoA was performed using the Bray‒Curtis distance. (S, T) Taxonomic cladograms were generated by LEfSe analysis. The blue color indicates enriched taxa in the VAMP group, and the red color indicates enriched taxa in the vehicle group. The size of each circle is proportional to the taxon's abundance. (U) The change in bacterial abundance at the species level after VAMP treatment was determined via random forest analysis. (V) Growth curve of *A. muciniphila* cultured in BHI media supplemented with VAMP (50 μM) or PBS (Vehicle) and then grown anaerobically. (W−Y) RNA‐seq analysis of the *A. muciniphila* cultured in BHI media supplemented with VAMP (50 μM) or PBS control (Vehicle) was performed (*n* = 3). (W) PCA of transcriptome. (X) Biofunction changes of *A. muciniphila* by VAMP treatment via KEGG enrichment analysis. (Y) Volcano plot of transcriptome sequencing of Vehicle and VAMP groups. (Z) Levels of peptidoglycan by VAMP treatment. *p < 0.05 and **p < 0.01 versus the vehicle group. AUC, area under the curve; BHI, brain heart infusion; FBG, fasting blood glucose; GLP‐1, glucagon‐like peptide‐1; HOMA‐IR, homeostatic model assessment of insulin resistance; IL‐1β, interleukin‐1β; LEfSe, linear discriminant analysis effect size; LPS, lipopolysaccharide; OGTT, oral glucose tolerance test; PCoA, principal coordinate analysis; TNF‐α, tumor necrosis factor α.

### Promotion of *Akkermansia muciniphila* growth by VAMP

Dysbiosis of gut microbiota is associated with the onset and progression of insulin resistance [9, 10], and we found that VAMP could alleviate insulin resistance and inflammatory response. We first determined the VAMP metabolic kinetics in model mice. VAMP showed a relatively low bioavailability (10.54%), and 74.18% of VAMP remained in the intestinal content after the gavage at the time of peak plasma concentration (Figure 2P,Q), suggesting that in addition to intestinal DPP‐IV inhibition, VAMP might affect the gut microbiota during the presence inside the intestinal lumen.

From the results of 16S rRNA gene sequencing, VAMP significantly altered the composition of colonic microbiota and increased the abundance of *A. muciniphila*, which was also negatively correlated with FBG and HOMA‐IR (Figure 2R−U and Figure S4A−G). Specifically, TPH also significantly increased the abundance of *A. muciniphila* in the intestine, whereas sitagliptin had no effect on the regulation of the bacteria (Figure S4H−P). We also validated that the *A. muciniphila*‐promotion and glucoregulation effects by VAMP treatment can be transferred by fecal microbiota transplantation (FMT) (Figure S5A−O).

### VAMP enables intestinal barrier function improvement to synergistically exert hypoglycaemic effects via specific promotion of *A. muciniphila* growth

As we found that VAMP increased the abundance of *A. muciniphila in vivo*, we investigated the influence of VAMP on the gut microbiota in vitro. In the faeces‐derived in vitro microbial community culture, VAMP was degraded by gut microbiota, and the abundance of *A. muciniphila* was significantly increased (Figure S6A−D). By incubating VAMP with different bacterial strains, we found that VAMP significantly increased the growth of *A. muciniphila*, *Bacteroides uniformis*, and *Lactiplantibacillus pentosus* (Figure 2V and Figure S6E). We also found that *A. muciniphila* could degrade VAMP, suggesting that VAMP could serve as a substrate or inducer for *A. muciniphila* growth (Figure S6F).

Next, we performed RNA sequencing analysis to investigate the potential mechanism underlying the VAMP‐mediated increase in *A. muciniphila*. Interestingly, the peptidoglycan biosynthesis pathway (for the synthesis of bacterial cell wall) was enriched by VAMP treatment via Kyoto Encyclopedia of Genes and Genomes enrichment analysis, as indicated by the upregulated expression of gene J4O16_RS10715 (K05366), which is associated with synthesis of transglycosylase domain‐containing protein that contributed to cell wall formation (Figure 2W−Z).

To investigate the role of *A. muciniphila* in glucose metabolism and insulin resistance during VAMP intervention, we developed an *A. muciniphila* “deletion” method [11]. We found that benzydamine hydrochloride (abbreviated as benzydamine) could specifically inhibit the growth of *A. muciniphila* (Figure S6G). We then treated the mice with benzydamine for the investigation of the role of VAMP on *A. muciniphila* (Figure S6H). VAMP treatment significantly increased the abundance of *A. muciniphila*, and benzydamine treatment reduced the abundance of *A. muciniphila* to an extremely low level (Figure S6I). VAMP improved glucose metabolism and intestinal barrier function, but these effects disappeared after simultaneous benzydamine administration (Figure S6J−Q). These results indicated that the specific growth‐promoting effects of VAMP on *A. muciniphila* are another important regulative effect of the peptide on glucose metabolism.

Food‐derived peptides are reported to have multiple biological functions, including the favorable DPP‐IV inhibitory activity, with fewer adverse effects than the synthetic drugs [12]. The traditional method for the mining of bioactive peptides requires complicated procedures such as separation, purification, identification, and synthesis of target peptides. We identified VAMP as a novel bioactive tetrapeptide with the strongest DPP‐IV inhibitory effects from TPH based on a combined high‐throughput screening method including multi‐omics, molecular docking, and machine learning toward a disease‐related target. The method can be used for the mining of biopeptides produced from various natural protein sources.

Peptides exert DPP‐IV inhibitory activity via competitive inhibition by binding at the catalytic site of DPP‐IV [13]. It has been identified that there are several subsites in DPP‐IV capable of binding competitively with inhibitors. They consist of two hydrophobic pockets, namely S1 and S2 pocket, mainly composed of amino acid residues such as ARG125, GLU205, GLU206, SER209, etc [14, 15]. Among which GLU205 and GLU206 play a crucial role in the recognition of substrate peptides, residues SER630 cleaves at the penultimate position of the N‐terminus [14]. Our study illustrated that VAMP could form hydrogen bonds with the DPP‐IV residues at ARG125, GLU205, GLU206, TYR547, SER630, TYR631, TYR662, and ASN710, and form salt bridges with the DPP‐IV residues at ARG125, GLU205, and GLU206 (Figure 1P, Q).

Manipulation of gut microbiota via dietary compounds might be a novel therapeutic strategy for metabolic diseases [16, 17]. *A. muciniphila* plays an important role in the maintenance of intestinal barrier integrity, thereby modulating the host inflammatory response [18]. The decreased abundance of *A. muciniphila* has been reported in numerous metabolic diseases. In the present study, both in vitro *A. muciniphila* fermentation experiments and in vivo animal experiments clearly showed that VAMP specifically promoted the growth of *A. muciniphila*. Peptidoglycans are the main components of bacterial cell wall. VAMP treatment upregulated the expression of gene J4O16_RS10715, which was responsible for the synthesis of penicillin‐binding protein 1A. Penicillin‐binding proteins catalyze the polymerization of the glycan strand (transglycosylation) and the cross‐linking between glycan chains (transpeptidation) and thus are indispensable for bacterial growth [19]. Therefore, VAMP might promote the growth of *A. muciniphila* by upregulation of peptidoglycan biosynthesis.

In conclusion, we developed an integrated mining method to discover novel DPP‐IV‐inhibitory bioactive peptides. VAMP from HSP exerted glucose‐lowering effects through fundamentally different mechanism via inhibition of DPP‐IV and specific promotion of the growth of intestinal *A. muciniphila*.

All the materials and methods, including the identification of bioactive peptides, molecular docking of the peptides with DPP‐IV, preparation of protein hydrolysates (Table S5), gene expression analysis (Table S6), and simulated digestive stability of the peptides, are described in the Supporting Information.

## AUTHOR CONTRIBUTIONS

**Haihong Chen**: Conceptualization; investigation; funding acquisition; writing—original draft; writing—review and editing; visualization; validation; methodology; software; formal analysis; data curation; project administration. **Wei Li**: Writing—original draft; writing—review and editing. **Wei Hu**: Methodology; validation. **Junyu Liu**: Methodology; validation. **Canyang Zhang**: Writing—review and editing. **Yi Wang**: Writing—review and editing. **Chong Zhang**: Writing—review and editing; conceptualization. **Xizhen Zhang**: Writing—review and editing. **Shuo Chen**: Writing—review and editing. **Qixing Nie**: Methodology; validation; investigation; conceptualization; visualization; writing—review and editing; writing—original draft; supervision; data curation; project administration. **Xinhui Xing**: Conceptualization; investigation; funding acquisition; writing—review and editing; supervision; resources.

## CONFLICT OF INTEREST STATEMENT

The authors declare no conflicts of interest.

## ETHICS STATEMENT

The ethics application (No. AEXXH202201) was approved by the Institutional Animal Care and Use Committee of Shenzhen Bay Laboratory.

## Supporting information


**Figure S1:** The enzymatic products of HSP processed by thermolysin inhibit DPP‐IV activity and improve glucose metabolism.
**Figure S2:** VAMP is an effective peptide inhibitor of DPP‐IV.
**Figure S3:** VAMP inhibits intestinal DPP‐IV and improves host glucose metabolism.
**Figure S4:** VAMP treatment increased the abundance of *A. muciniphila*.
**Figure S5:** VAMP treatment improved gut microbiota and glucoregulatory effects can be transferred via FMT.
**Figure S6:** VAMP improves host glucose metabolism by promoting the expansion of *A. muciniphila*.

**Table S1:** Amino acids composition of HSP.
**Table S2:** Unigene IDs of the identified proteins.
**Table S3:** Values of relative response, KD values, and IC_50_.
**Table S4:** Detail interaction information of DPP‐IV and VAMP.
**Table S5:** Conditions for protein hydrolysis.
**Table S6:** Primer sequences for RT‒qPCR.

## Data Availability

The 16S rRNA gene sequencing raw sequence reads (fastq) and RNA‐seq sequencing data produced in this study are available at the NCBI Sequence Read Archive with the BioProject: PRJNA1234239 (https://www.ncbi.nlm.nih.gov/bioproject/PRJNA1234239) and BioProject: PRJNA1234295 (https://www.ncbi.nlm.nih.gov/bioproject/PRJNA1234295). Crystal structure of DPP‐IV‐VAMP has been validated at the Worldwide Protein Data Bank (wwPDB), and the accession code was 9LBT (https://doi.org/10.2210/pdb9lbt/pdb). The data and scripts used in this study are available on GitHub at https://github.com/chenchen12345/CHH_DPP_IVi_202507/tree/main. Supplementary materials (methods, figures, tables, graphical abstract, slides, videos, Chinese translated version, and update materials) may be found in the online DOI or iMeta Science http://www.imeta.science/.
