# Supplementary material for: Discovery of a novel tetrapeptide as glucose homeostasis modulator with bifunctionalities of targeting DPP‐IV and microbiota
Source: Imeta. 2025 Aug 11;4(5):e70072. doi: 10.1002/imt2.70072 (PMC12527977; doi:10.1002/imt2.70072)
Supplement: Supplementary file 1 — Figure S1: The enzymatic products of HSP processed by thermolysin inhibit DPP‐IV activity and improve glucose metabolism. Figure S2: VAMP is an effective peptide inhibitor of DPP‐IV. Figure S3: VAMP inhibits intestinal DPP‐IV and improves host glucose metabolism. Figure S4: VAMP treatment increased the abundance of A. muciniphila. Figure S5: VAMP treatment improved gut microbiota and glucoregulatory effects can be transferred via FMT. Figure S6: VAMP improves host glucose metabolism by promoting the expansion of A. muciniphila. [file IMT2-4-e70072-s001.docx]

**Supporting information to**

**Discovery of a novel tetrapeptide as glucose homeostasis modulator with bifunctionalities of targeting DPP-IV and microbiota**

**Running title**: hemp seed proteins-derived VAMP bifunctionally improves glucose homeostasis

Haihong Chen^1, 2^, Wei Li^1, 2^, Wei Hu^1^, Junyu Liu^1^, Canyang Zhang^1, 2, 3^, Yi Wang^3, 4^, Chong Zhang^3, 4^, Xizhen Zhang^5^, Shuo Chen^6^, Qixing Nie^7*^, Xinhui Xing^1, 2, 3, 4^*

^1^Institute of Biopharmaceutical and Health Engineering, Tsinghua Shenzhen International Graduate School, Shenzhen 518055, China

^2^Key Laboratory of Active Proteins and Peptides Green Biomanufacturing of Guangdong Higher Education Institutes, Tsinghua Shenzhen International Graduate School, Shenzhen 518055, China

^3^Key Laboratory for Industrial Biocatalysis, Ministry of Education, Institute of Biochemical Engineering, Department of Chemical Engineering, Beijing 100084, China

^4^Center for Synthetic and Systems Biology, Tsinghua University, Beijing 100084, China

^5^College of life Science and Technology, Guangxi University, Nanning, 530004, China

^6^Department of Psychiatry, New York University Grossman School of Medicine, New York, 10016, United States

^7^State Key Laboratory of Food Science and Resources, China-Canada Joint Lab of Food Science and Technology, Key Laboratory of Bioactive Polysaccharides of Jiangxi Province, Nanchang University, Nanchang, 330047, China

*Correspondence: [xhxing@tsinghua.edu.cn](mailto:xhxing@tsinghua.edu.cn) (Xinhui Xing); [qixingnie@ncu.edu.cn](mailto:qixingnie@ncu.edu.cn) (Qixing Nie)

**METHODS**

**Chemicals and reagents**

Human DPP-IV (IBDPP0901) was obtained from QiZheng Biotech (Shanghai, China). The DPP-IV-Glo^TM^ Protease Assay Kit (G8351) was purchased from Promega (Madison, Wisconsin). An active GLP-1 ELISA kit (EGLP-35K) was purchased from Millipore (Billerica, Massachusetts). Total GLP-1 (CB10786-Mu), LPS (CB10838-Mu), TNFα (CB10851-Mu), IL-1β (CB10173-Mu), and NEFA (CB10282-Mu) kits were purchased from COIBO BIO Technology (Shanghai, China). The peptides VFTPQ, VADW, VAMP, FNPRG, FNVDSE, FPQS, FQL, FLQ, WINVN, WIAVK, PSSQQ, YTPHW, YSYA, YTGD, YQLM, FDGEL, PQNHA, LNAP, YNLP, YQL, LLY, WDSY, WLE, and YNL (> 98% purity) were synthesized by Bankpeptide Inc. (Hefei, China). Series S sensor chips (CM5), HEPES buffer (SH30237.01), an amine coupling kit (BR100050), 50 mM NaOH (BR100358), and 10 mM glycine (BR100354) were obtained from GE Healthcare Life Sciences (Uppsala, Sweden). Sitagliptin (S4002) was purchased from Selleck (Houston, TX). Krebs-Ringer bicarbonate-HEPES buffer (KRBH, R30083) was obtained from Shanghai Yuanye Bio-Technology Co., Ltd. (Shanghai, China).

**Nutritional composition analysis of hemp seeds**

Hemp seeds (*Cannabis sativa* L.) were obtained from Bama Miao Autonomous County, [the Guangxi Zhuang Autonomous Region](http://www.baidu.com/link?url=oKxbf6g8AuycGO4GtrXMNroNfFtgFxu1jBJE8qD56frWTfBuzvGW5j3EGeAZ8BHe7Ci3WHlK8TIvVtMRXhPU9F1qCdtHEMTOiCBzD7DA9L9899xvddrJ7JyVTvJVooIoJgWPdvRBQg3NvFWjqxPsnK), China. The dietary fiber, moisture, ash, protein, fat, and amino acid composition of the hemp seeds were determined according to the Chinese National Standard GB 5009.88-2014, GB 5009.3-2016, GB 5009.4-2016, GB 5009.5-2016, GB 5009.6-2016, and GB 5009.124-2016, respectively.

**Functional annotation of hemp seed peptides**

The simulation of the protease cleavage sites of hemp seed proteins and peptide functional annotation were performed according to our previous methods [1]. In brief, 1184 proteins identified in our previous study were imported into PeptideMass (https://web.ExPASy.org/peptide_mass/), and simulation of the protein cleavages were performed with trypsase, chymotrypsin, trypsase/chymoptrypsin, pepsase, proteinase K, and thermolysin. The cleaved peptides were selected in the range of 0−3000 Da. Following functional annotation, the peptides were imported into the BIOPEP-UWM server (<http://www.uwm.edu.pl/biochemia/index.php/pl/biopep/)>.

**Preparation of protein hydrolysates**

The preparation of protein hydrolysates was performed according to our previous methods [2]. Briefly, hemp seed oil was removed with ethanol, protein was extracted with 0.8 mol/L NaCl aqueous solution (pH 7.0), then the enzymatic hydrolysis of extracted hemp seed protein was performed, and the details of the enzymatic hydrolysis conditions are listed in Table S5.

**Molecular weight distribution of HSP hydrolysates**

The homogeneity and distribution of the enzymolysis solutions were evaluated using high-performance gel permeation chromatography (HPGPC) according to our previous method [2]. In brief, an HPLC instrument (Agilent 1260) equipped with a TSK-Gel G2000SWXL (300 mm × 7.8 mm) column was used for data acquisition. The mobile phase was acetonitrile:water:trifluoroacetic acid (30:70:0.1), the wavelength was set at 220 nm, the flow rate was 0.5 mL/min, and the column temperature was 30 °C. Cytochrome C, aprotinin, VPSGPLGPEGPR, glutathione, and glutamate were used as standards to plot a standard curve under the described chromatographic conditions.

**Evaluation of DPP-IV inhibition by HSPs hydrolysates and synthetic peptides using iPSC-induced intestinal organoids**

iPSC-induced intestinal organoids were established according to the manufacturer’s instructions (STEMdiff^TM^ Intestinal Organoid kit). After the intestinal organoids had fully established (passaged at 3−10 generations), the organoids were treated with 500 μg/mL of hydrolysates, 10 μM sitagliptin or varied concentration of synthetic peptides (100, 200, 500 μg/mL) for 24 h, the mixtures were centrifuged at 500 × g for 5 min, and the supernatants were used to determine the inhibition of DPP-IV and the concentrations of active GLP-1 by using the DPP-IV-Glo^TM^ Protease Assay Kit and GLP-1 ELISA Kit, respectively.

**Identification of bioactive peptides in TPH**

To identify bioactive peptides in TPH, the < 3 kDa fraction of TPH was collected. A nanoLC-MS/MS (Thermo Scientific, Waltham, MA) was used for raw data acquisition. Briefly, peptides were dissolved in mass spectrometry loading buffer and separated on a Waters ACQUITY UPLC® BEH C18 column (2.1 mm × 50 mm, 1.7 μm particle size). The gradient elution consisted of 0.1% formic acid in water (solution A) with formic acid in acetonitrile (solution B) as the mobile phase at a flow rate of 250 nL/min. The gradient program for the binary mobile phase system was as follows: 0−2 min, 3−7% B; 2−52 min, 7−22% B; 52−62 min, 22−35% B; 62−64 min, 35−90% B; and 64−84 min, 90−90% B. The m/z scan range was 200 to 2000. MaxQuant 1.5.3.17 software was used for raw data processing.

**Molecular docking of the peptides with DPP-IV**

Molecular docking was performed according to our previously methods [2]. In details, the potential binding capacity between hDPP-IV and the peptides was evaluated using PyRx 0.9 software (Autodock 4.0). The three-dimensional (3D) crystal structures of DPP-IV-IPI (1NU8) complexes were downloaded from the Protein Data Bank (https://www.rcsb.org/), and the protein receptors were configured for binding using PyMOL (version 2.4) by removing the B/C/D chains, ligands, and water molecules. MGL Tools software (version 1.5.6) was used to prepare the PDBQT format files for the in-silico docking of receptors (proteins) and ligands (used for grid box generation). The interaction between the receptors and ligands was performed using PyRx software (version 0.9). The centres of the grid box were assigned to 1NU8 (X=68.5567, Y=73.1337, Z=67.8251), 3WQH (X=44.8455, Y=49.5603, Z=28.6399), 4J3J (X=5.8263, Y=18.2281, Z=-22.3054), 4PNZ (X=40.9980, Y=50.8154, Z=37.7065), and 5YP3 (X=10.3137, Y=-52.8368, Z=-132.3067). The number of points in the box were X:50, Y:50, and Z:50, and the spacing of the box was 0.3750 Å. The DPP-IV-peptide interactions were evaluated based on their binding affinity. We also applied 30 different initial conformations to assess reproducibility, with binding energy was employed for the scoring.

**Machine learning-based pIC_50_ prediction of peptides**

A sample two-layer neural network was built to obtain the predicted pIC_50_ values. Briefly, a subset of the dataset containing 3681 DPP-IV inhibitors with validated IC_50_ values was acquired from the ChEMBL database (https://www.ebi.ac.uk/chembl/). Then, we convert the molecules to Molecular ACCess System fingerprints (MACCS fingerprints) to apply a neural network according to the Keras workflow, which included preparing the data, defining the model, compiling the model, fitting the model, and evaluating the model and predictions the pIC_50_ values of the peptides. The mean squared error (MSE) and mean absolute error (MAE) were used for the evaluation of the established model [3−5].

**Selection of potential bioactive peptides**

To screen the potential bioactive peptides, dynamic network Venn diagram analysis was performed for the top 100 peptides (tripeptides, tetrapeptides, pentapeptides, and hexapeptides) in TPH based on the intensity, affinity, and predicted pIC_50._

**Evaluation of DPP-IV inhibitory activity for the selected peptides**

A DPP-IV-Glo^TM^ Protease Assay Kit was used to determine the inhibitory effect of the peptides on DPP-IV. The experiment was performed according to the instructions. The mode of DPP-IV inhibition was analysed according to our previously methods [1].

**Surface plasmon resonance (SPR)**

The affinity between DPP-IV and the selected peptides was evaluated by using a Biacore 8K high-throughput intermolecular interaction analysis system (GE Healthcare, Chicago, IL) with CM5 chips ([Cytiva](http://www.baidu.com/link?url=mKgAvPy7j6G5wrpl9U5mRitIRGsXasLWtax8RKodqmEJZ8RPT9Xtwphfs8_1Ats8L5sME9bFsfevx1xDqFH4Lq), Marlborough, MA) at 25 °C. The specific experimental processes were performed according to our previous study [1].

**Simulated digestive stability of peptides**

The *in vitro* digestibility of the peptides was determined according to the method described by Ohanenye *et al*. with some modifications [6]. Briefly, 5.0 mg/mL peptides were mixed with 5000 U/mL porcine pepsase (pH 3.0). The mixture was incubated with continuous shaking in a water bath at 37 ℃ for 2 h. Then, the mixture was adjusted to pH 7.0, and a final concentration of 100 U/mL trypsase was added, followed by continuous shaking at 37 ℃ for another 2 h. After digestion, the enzyme was inactivated, and the mixture was incubated in a water bath (100 ℃) for 20 min. Subsequently, the mixture was centrifuged at 6000 rpm for 20 min, after which the supernatants were collected for further analysis. The concentrations of peptides in the digestions and TPH were determined via AB SCIEX QTrap 6600+ (AB Sciex, Framingham, Massachusetts).

**Crystallization and structure determination of VAMP-DPP-IV**

Crystallization and structure analyzed of VAMP-DPP-IV were performed at Technology center for protein sciences, Tsinghua university, including protein expression and purification, crystallization, data Collection, and structure determination. The ectodomain of human DPP-IV (residues 33-766) were cloned into the pFastBac-dual vector for baculovirus expression (Bac-to-Bac baculovirus expression system, Invitrogen). an N-terminal Hemolin signal peptide for secretion and a C-terminal hexa-His were added to facilitate protein secretion and purification. The recombinant plasmid was transformed into DH10Bac competent cells to get the recombinant Bacmid. Then the Bacmid was transfected into Sf9 to get the baculovirus stock. Sf9 was then used to amplify the baculoviruses, and Sf9 cells were used to express the protein. The supernatant of Sf9 was collected 48 h post-infection and passed through a 5 mL HisTrap HP column (GE Healthcare, Milwaukee, Wisconsin). The proteins bound to HisTrap were then detached by gradient concentrations of imidazole. The fractions containing the target protein was determined by SDS-PAGE. The samples were then pooled and further purified by a Superdex 200 increase column (GE Healthcare) with the buffer of 71 mM Gly–NaOH buffer pH 8.7. The proteins were then used for crystallization. For protein crystallization, DPP-IV was mixed with VAMP at a 1:10 stoichiometry and crystallized by the hanging-drop vapor diffusion method at 16 ^o^C at 15 mg/mL in a buffer consisting of 0.25 M Ammonium acetate, 0.1 M Tris pH 8.5, 21% w/v Polyethylene glycol 3350. Diffraction data were collected with cryoprotected (in a reservoir solution containing 20% [v/v] glycerol) crystals at the Shanghai Synchrotron Radiation Facility (SSRF) BL02U1. The diffraction data were processed and scaled with HKL-2000. Molecular replacement was performed with Phaser in PHENIX and the structure was further built manually with Coot and refined with PHENIX.Refine.

**Animals and peptide treatments**

All our animal experiments were approved by the Shenzhen Bay Laboratory (permit: AEXXH202201). C57BL/6J mice were purchased from Beijing Vital River Laboratory Animal Technology Co., Ltd. (Beijing, China). *ob/ob* mice (T001461) were purchased from GemPharmatech Laboratory (GemPharmatech, Nanjing, China). Mice were maintained under a strict 12 h light cycle and had unlimited access to water and food. All mice were randomly assigned to the experimental groups; the groups did not present differences in body weights before the treatments, and no mice were excluded from the analysis.

To evaluate the hypoglycaemic activity of TPH, all mice were fed a high-fat diet (HFD, Research Diets, cat# D12492). Eight-week-old C57BL/6J SPF mice were supplemented daily with PBS (HFD group), sitagliptin (12.5 mg/kg, Sit group), hemp seed protein hydrolysates by thermolysin (80 mg/kg, TPH group), and hemp seed protein (320 mg/kg, HSP group) for eight weeks by oral gavage.

To test the *in vivo* effect of VAMP on host DPP-IV, eight-weeks-old C57BL/6J SPF mice were fed a HFD for eight weeks, then treated daily with VAMP (50 mg/kg) or PBS (vehicle group) for one week.

To investigate the effects of VAMP on glucose metabolism, eight-weeks-old C57BL/6J SPF mice were treated daily with PBS (vehicle group) or VAMP (50 mg/kg, VAMP group) for eight weeks by oral gavage.

To test the hypoglycaemic and DPP-IV inhibitory effects of VAMP in *ob/ob* mice, 6-week-old *ob/ob* mice were treated daily with PBS (vehicle group) or VAMP (50 mg/kg, VAMP group) for four weeks by oral gavage.

For FMT experiment, mice were treated with Abx for one week. Feces from mice treated with VAMP or PBS were collected. Feces (100 mg) were resuspended in sterile anaerobic PBS (1 mL) and then centrifuged at 200 × g for 3 min at 4 °C, the supernatant was collected and administered to mice after Abx treatment.

To investigate the effects of *A. muciniphila* in improving glucose metabolism during VAMP treatment, mice were fed a HFD for eight weeks. Then mice were treated with PBS, 50 mg/kg VAMP, 50 mg/kg benzydamine hydrochloride, or 50 mg/kg VAMP combined with benzydamine hydrochloride three times per week for four weeks.

**Metabolic assays**

Oral glucose tolerance tests (OGTTs) were conducted following a 6-hour fasting period in mice. Blood glucose levels were assessed using a glucometer on tail vein blood samples at 0, 15, 30, 60, and 90 min after oral glucose administration with a dosage of 1.5 g/kg body weight. For insulin tolerance tests (ITTs), mice were intraperitoneal injected with insulin (0.8 U/kg body weight) after 6 h of fasting. For measurement of active GLP-1 (Millipore, cat# EGLP-35K), total GLP-1 (Elabscience, cat# E-EL-M0090c), and insulin (ABclonal, cat# RK02951), blood was collected before gavage and 15 min after glucose gavage; the blood was supplemented with 10 mM sitagliptin, and the plasma was stored at -80 ℃ until further analysis. To measure the indicators in intestinal tissue, a 0.5-cm segment of distal gut tissue was homogenized in RIPA buffer supplemented with 10 mM sitagliptin. The levels of active GLP-1, total GLP-1, and insulin were measured using the corresponding kits and normalized to the protein concentration (via a BCA protein assay).

**Plasma biochemical analysis**

The levels of plasma total cholesterol (TC), triacylglycerol (TG), high-density lipoprotein cholesterol (HDL-c), low-density lipoprotein cholesterol (LDL-c), nonesterified fatty acid (NEFA), tumour necrosis factor α (TNF α), and interleukin-1β (IL-1β) were determined by commercial kit (Nanjing Jiancheng Bioengineering Institute, Nanjing, China).

**Intestinal permeability analysis**

Intestinal permeability was evaluated through the oral administration of fluorescein-isothiocyanate (FITC)-dextran in mice that were subjected to a 4-hour fasting period. Following the gavage of FITC-dextran at a dose of 200 mg/kg body weight, blood samples were collected from the tail vein after 90 min. The collected blood was centrifuged at 3000 × *g* for 10 min to obtain serum samples. Subsequently, 20 μL aliquots of serum were plated in 96-well plates, diluted with PBS to a final volume of 200 μL, and analyzed for fluorescence intensity at excitation and emission wavelengths of 485 nm and 520 nm, respectively.

**Gene expression analysis**

Mouse tissues were cryopreserved in liquid nitrogen at -80 °C, followed by standard phenol‒chloroform extraction using TRIzol reagent to isolate total RNA. Subsequently, cDNA was synthesized from 2 μg of total RNA using a reverse transcription kit. The quantification of individual mRNA levels was determined by normalizing to β-actin mRNA.

Reverse transcription of total RNA was performed using the PrimeScript RT reagent kit (Takara). Quantitative real-time PCR was performed using TB Green Premix Ex Taq II (Takara) on a QuantStudio 7 Flex Real-Time PCR system (Thermo Fisher Scientific, Waltham, Massachusetts). The primers were listed in the Key Resources Table. β-actin was used as an endogenous control. The relative mRNA expression levels were calculated using the 2^-△△Ct^ quantification method.

Mouse tissues were frozen and stored at -80 °C, then RNA was extracted from the tissue using RNA isolation Kit. cDNA was synthesized using QuantiTect Reverse Transcription kits. Quantitative real-time PCR was performed using TB Green Premix Ex Taq II (Takara) on a QuantStudio 7 Flex Real-Time PCR system (Thermo Fisher Scientific, Waltham, Massachusetts). β-actin was used as an endogenous control. The relative mRNA expression levels were calculated using the 2^-△△Ct^ quantification method. The sequences of primers used for RT‒qPCR are shown in Table S6.

**Histological analysis**

For histological analysis of subcutaneous white adipose tissue, subsections were partially embedded in 10% neutral buffered formalin solution (Sigma, St. Louis, Missouri). Paraffin-embedded adipose tissue sections were stained with haematoxylin and eosin (H&E) for morphological examination. Changes in histological fat sections were observed under a light microscope, and the cell area was calculated with ImageJ software (US National Institutes of Health, Bethesda, Maryland).

For histological analysis of colonic tissue, colon segments were promptly immersed in Carnoy’s fixative for 24 h. Subsequently, the colon specimens underwent standard dehydration procedures before being embedded in paraffin, and thin sections (5 μm) were cut and deposited on glass slides. The paraffin sections were stained with Alcian blue/periodic acid-Schiff. The thickness of the colonic sections was then measured by ImageJ, and five different measurements were made perpendicular to the inner mucus layer per image. Only regions in which the mucus layer was sandwiched between the epithelium on one side and luminal contents on the other were used; care was taken to measure regions that represented the average thickness in each blinded image.

**Gut microbiota analysis**

Total DNA was extracted from colonic contents using a Tiangen stool DNA extraction kit. The V3−V4 region of the 16S rRNA gene was amplified by PCR from the extracted and purified genomic DNA using 515 forward and 806 reverse primer pairs. PCR amplification was performed on a PCR System (Bio-Rad, Hercules, California), and the PCR amplification products were separately extracted from a 2% agarose gel and further purified using a Tiangen agarose gel DNA purification kit. The purified amplicons were quantified using a Qubit Fluorometer (Thermo Fisher Scientific, Waltham, Massachusetts), pooled in equimolar, and sequenced on an Illumina Miseq platform (Illumina, San Diego, California).

Raw sequencing data were processed with QIIME2. In brief, the forward and reverse reads each were truncated at 200 bases. Taxonomy was assigned using the Greengenes reference (version 13.8) database. Analyses of alpha diversity (one-way ANOVA followed by Tukey’s post hoc test), beta diversity (weighted UniFrac distance followed by ANOSIM test), and bacterial taxonomic distributions were performed using MicrobiomeAnalyst.

**Stability of the VAMP**

The faeces-derived bacterial community or *A. muciniphila* were incubated in BHI medium supplemented with 100 μM VAMP for 48 h. The levels of VAMP at different time points were quantified by targeted metabolomics.

**Statistical analysis**

All the statistical data were analysed using SPSS version 26.0. All experimental data are reported as the mean ± SEM. Two-tailed unpaired Student's t-test or one-way ANOVA for multiple comparisons followed by Tukey’s test was used to determine the significance of differences. *p* < 0.05 was considered significant.

**Supplementary References**

1. Chen, Haihong, Wei Li, Yi Wang, Bing Xu, Xi Hu, Xiaobing Li, Junyu Liu, Chong Zhang, Canyang Zhang, Xinhui Xing. 2023. “Mining and validation of novel Hemp Seed-derived DPP-IV-inhibiting peptides using a combination of multi-omics and molecular docking.” *Journal of Agricultural and Food Chemistry* 71: 9164-9174. <https://doi.org/10.1021/acs.jafc.3c00535>

2. Li, Wei, Haihong Chen, Hongliang Chen, Ziyin Li, Wei Hu, Qinxuan Zhou, Bing Xu, Yi Wang, Xinhui Xing. 2024. “*Andrias davidianus* bone peptides alleviates hyperuricemia-induced kidney damage *in vitro* and *in vivo*.” *Food Science and Human Wellness* 13: 1886-1905. <https://doi.org/10.26599/FSHW.2022.9250157>

3. Amari, Shun-ichi. 1993. “Backpropagation and stochastic gradient descent method.” *Neurocomputing* 5: 185-196. [https://doi.org/10.1016/0925-2312(93)90006-O](https://doi.org/https://doi.org/10.1016/0925-2312(93)90006-O)

4. Amato, Filippo, Alberto López, Eladia María Peña-Méndez, Petr Vaňhara, Aleš Hampl, Josef Havel. 2013. “Artificial neural networks in medical diagnosis.” *Journal of Applied Biomedicine* 11: 47-58. [https://doi.org/10.2478/v10136-012-0031-x](https://doi.org/https://doi.org/10.2478/v10136-012-0031-x)

5. Sydow, Dominique, Andrea Morger, Maximilian Driller, Andrea Volkamer. 2019. “TeachOpenCADD: a teaching platform for computer-aided drug design using open source packages and data.”*Journal of Cheminformatics* 11: 29. <https://doi.org/10.1186/s13321-019-0351-x>

6. Ohanenye, Ikenna Christian, Xiaohong Sun, Roghayeh Amini Sarteshnizi, Chibuike C Udenigwe. 2021. “Germination alters the microstructure, in vitro protein digestibility, α-glucosidase and dipeptidyl peptidase-IV inhibitory activities of bioaccessible fraction of pigeon pea (*Cajanus cajan*) seeds.” *Legume Science* 3: e79. <https://doi.org/10.1002/leg3.79>


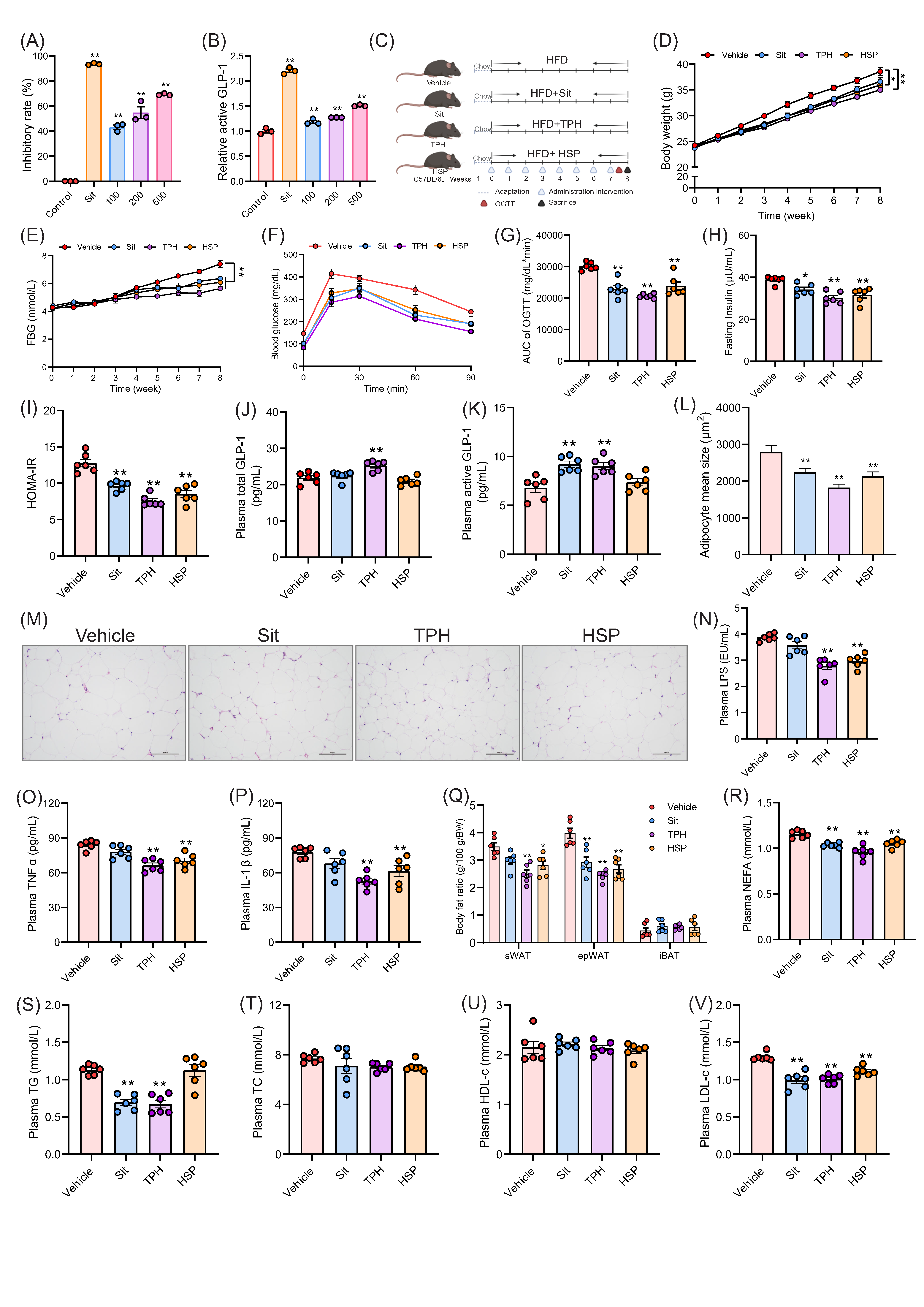


**Figure S1 The enzymatic products of HSPs processed by thermolysin inhibit DPP-IV activity and improve glucose metabolism.** (A) The inhibitory effect of different concentrations of TPH on DPP-IV activity was measured. (B) The relative concentrations of active GLP-1 after TPH treatment in intestinal organoids were determined. (C) The experimental scheme for (D to V) is shown, *n* = 6 mice/group. HFD-fed mice were treated with PBS (vehicle group), sitagliptin (sit group), HSPs hydrolysates by thermolysin (TPH group), or HSPs (HSP group) for 8 weeks by oral gavage. (D) The body weight change, (E) FBG, (F) OGTT curve, (G) AUC of the OGTT, (H) levels of fasting insulin, (I) HOMA-IR index, (J) levels of plasma total GLP-1, and (K) levels of plasma active GLP-1 were determined. (L) The mean adipocyte size (3 mice per group). (M) Representative H&E images of sWAT deposits (3 mice per group) are shown. Scale bars, 100 μm. (N) plasma LPS level, (O) plasma TNFα level, and (P) plasma IL-1β level were determined. (Q) The weights of sWAT, epWAT, and iBAT. (R−V) The levels of plasma NEFAs, TG, TC, HDL-c, LDL-c. All the data are presented as the mean ± SEM. **p* < 0.05 and ***p* < 0.01 versus the vehicle group. DPP-IV, dipeptidyl peptidase IV; GLP-1, glucagon-like peptide-1; HSP, hemp seed proteins; TPH, thermolysin-catalyzed protein hydrolysates; OGTT, oral glucose tolerance test; AUC, area under the curve; HOMA-IR, homeostatic model assessment of insulin resistance; TNFα, tumor necrosis factor α; IL-1β, interleukin-1β; LPS, lipopolysaccharide; NEFAs, non-esterified fatty acids; TG, total triglycerides; TC, total cholesterol; HDL-c, high-density lipoprotein cholesterol; LDL-c, low-density lipoprotein cholesterol.


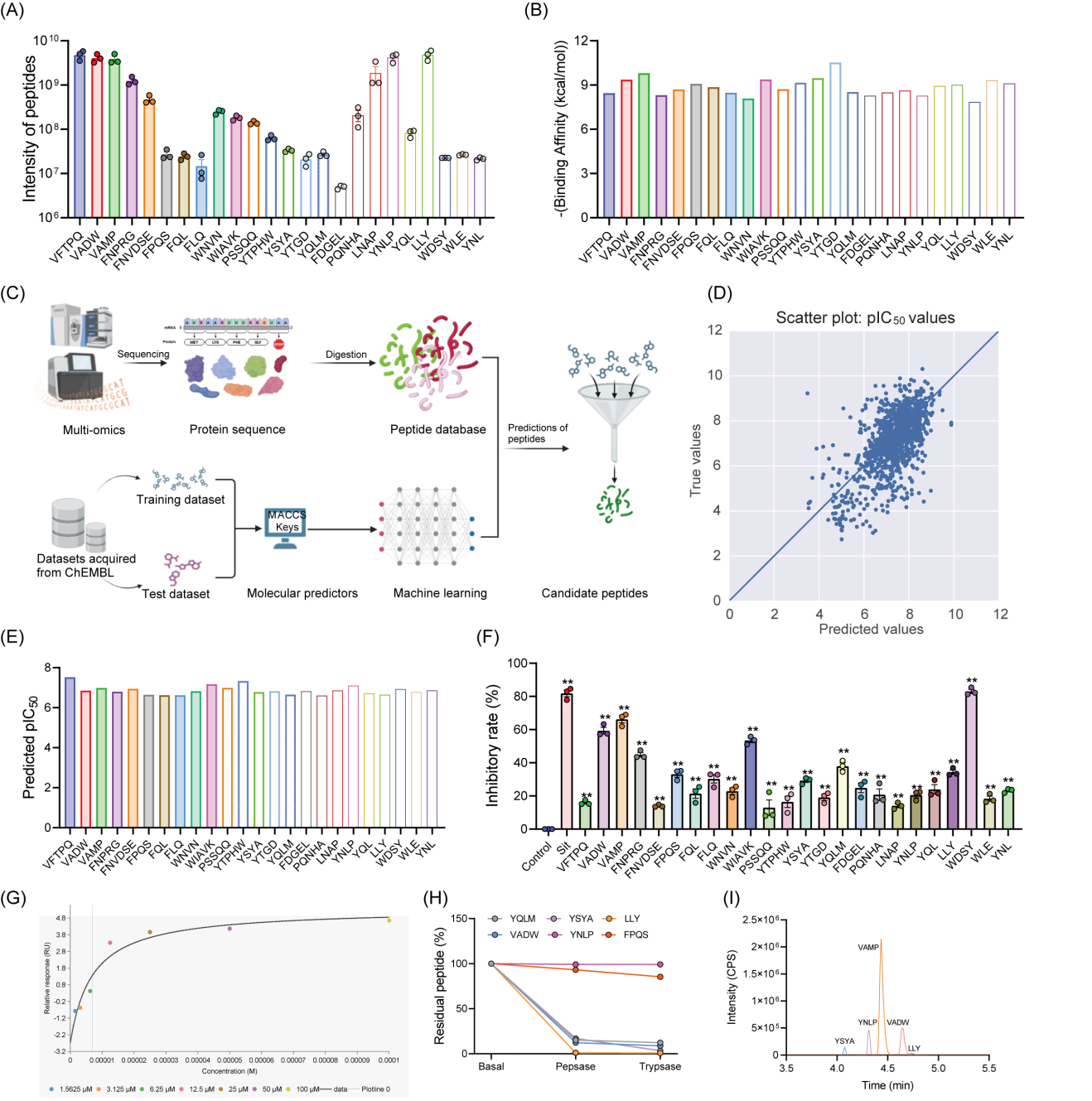


**Figure S2 VAMP is an effective peptide inhibitor of DPP-IV**. (A) The relative intensity of the 24 selected peptides in the TPH sample is shown. (B) PyRx-Dock-derived docking binding affinity for DPP-IV (receptor) and the 24 selected peptides. (C) Strategy for machine learning. (D) Scatter plot for visualization of the correlation between the predicted and true pIC_50_ values in the test set. (E) Predicted pIC_50_ values of the 24 selected peptides. (F) DPP-IV inhibition by the 24 selected peptides. (G) The fitting curves for the sensorgram. (H) The residual VADW, YQLM, YSYA, LLY, FPQS, and YNLP after pepsase and trypsase digestion are shown. (I) Representative extracted ion chromatograms of YSYA, YNLP, VAMP, VADW, and LLY in the TPH sample are shown. TPH, thermolysin-catalyzed protein hydrolysates; pIC_50_, predicted half maximal inhibitory concentration; DPP-IV, dipeptidyl peptidase IV.


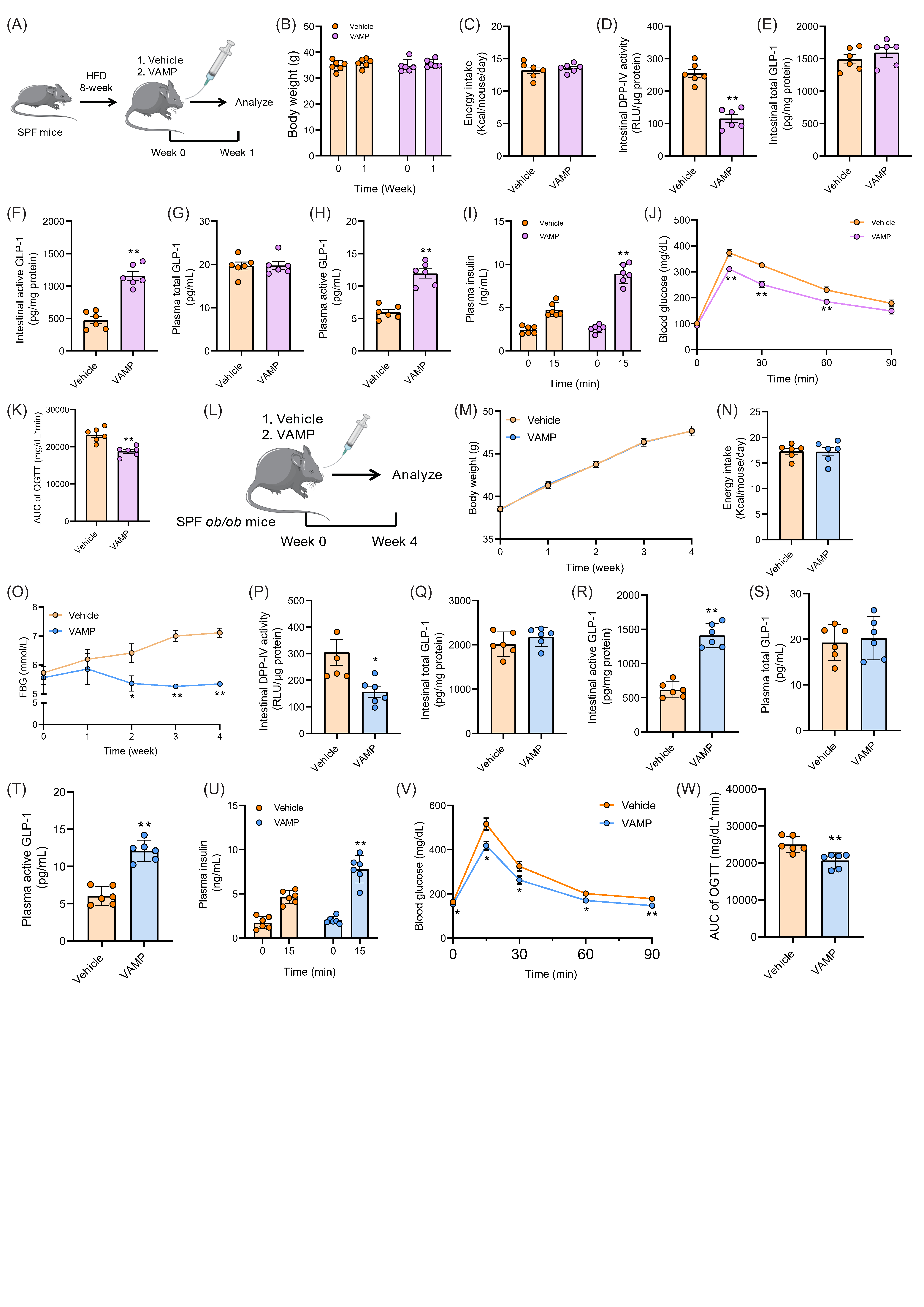


**Figure S3 VAMP inhibits intestinal DPP-IV and improves host glucose metabolism.** (A) The experimental scheme for (B to K) is shown, *n* = 6 mice/group. Mice were fed a HFD for 8 weeks, divided into two groups, and treated with PBS (vehicle group) or VAMP (50 mg/kg, VAMP group) for 1 week. (B) The body weights, (C) energy intake, (D) DPP-IV activity in intestinal tissue, (E) intestinal total GLP-1 levels, (F) intestinal active GLP-1 levels, (G) plasma total GLP-1 levels, (H) plasma active GLP-1 levels, (I) glucose-stimulated insulin levels, (J) OGTT curve, and (K) AUC of the OGTT were determined. (L) The experimental scheme for (M to W) is shown, *n* = 6 mice/group. The *ob*/*ob* mice were divided into two groups and treated with PBS (vehicle group) or VAMP (50 mg/kg, VAMP group) for 4 weeks by oral gavage. (M) Body weight changes, (N) energy intake, (O) changes in FBG levels, (P) DPP-IV activity in intestinal tissue, (Q) intestinal total GLP-1 levels, (R) intestinal active GLP-1 levels, (S) plasma total GLP-1 levels, (T) plasma active GLP-1 levels, (U) glucose-stimulated insulin levels, (V) OGTT curve, and (W) AUC of the OGTT were evaluated. All the data are presented as the mean ± SEM. **p* < 0.05 and ***p* < 0.01 versus the vehicle group. DPP-IV, dipeptidyl peptidase IV; GLP-1, glucagon-like peptide-1; OGTT, oral glucose tolerance test; AUC, area under the curve.


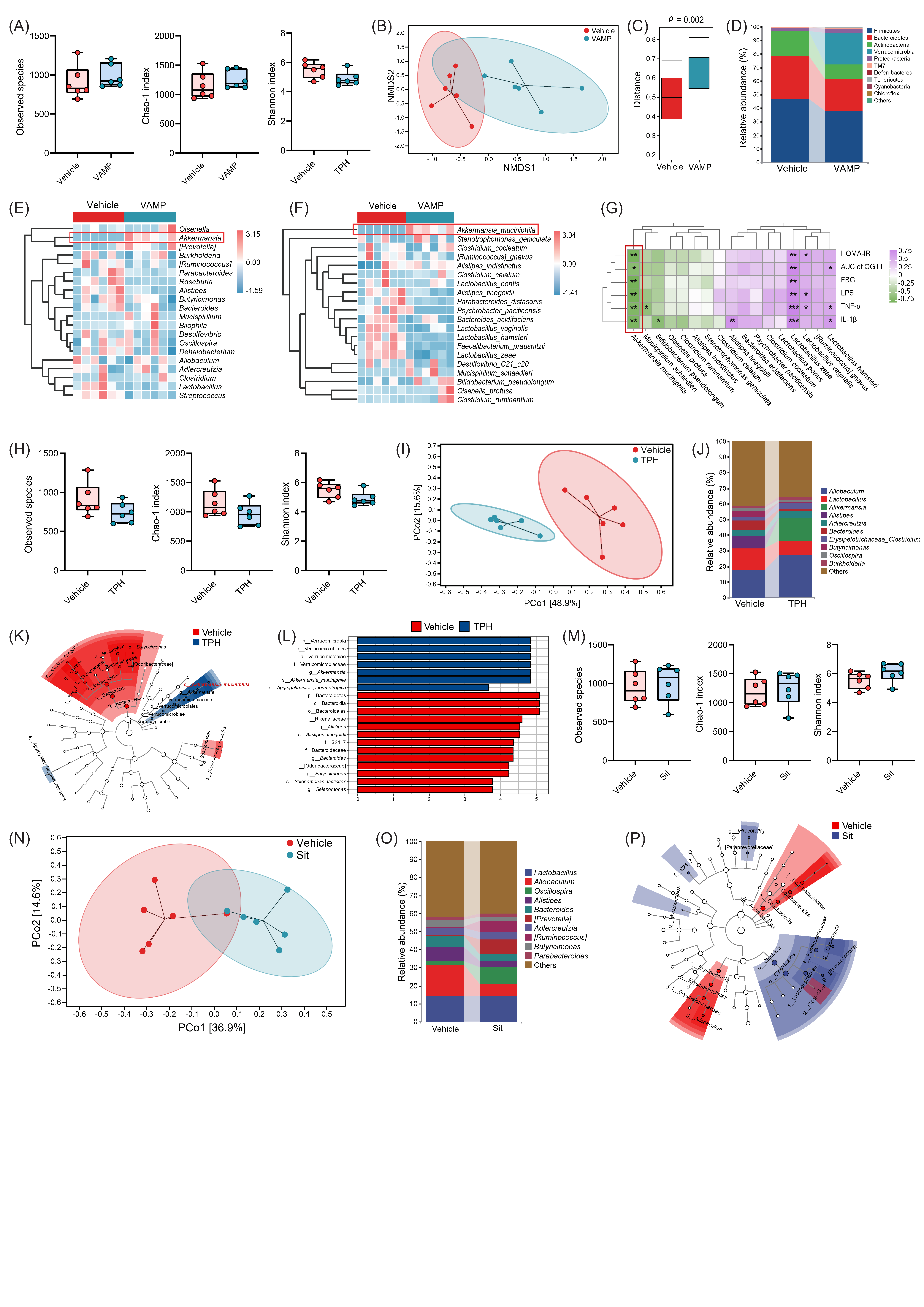


**Figure S4 VAMP treatment increased the abundance of *A. muciniphila***. (A) The α diversity of the gut microbiota was compared between the vehicle and VAMP groups. (B) NMDS analysis was performed. (C) The Bray‒Curtis distance. (D) Phylum-level compositions of the gut microbiota in the vehicle and VAMP groups. (E) A heatmap of the gut microbiota at the genus level. (F) A heatmap of the gut microbiota at the species level. (G) Correlative analysis between gut microbiota and glucose metabolism related parameters. (H−J) HFD-fed mice were treated with PBS (vehicle group) or TPH (TPH group) for 8 weeks by oral gavage. (H) α diversity. (I) PCoA was performed using the Bray‒Curtis distance. (J) The genus-level differences in the composition of the gut microbiota between the vehicle and TPH groups were evaluated. (K and L) Taxonomic cladograms were generated by LEfSe analysis. (M−P) HFD-fed mice were treated with PBS (vehicle group) or sitagliptin (sitagliptin group) for 8 weeks by oral gavage. (M) α diversity. (N) PCoA was performed using the Bray‒Curtis distance. (O) The genus-level composition of the gut microbiota. (P) Taxonomic cladograms were generated by LEfSe analysis. NMDS, nonmetric multidimensional scaling; HFD, high fat diet; PCoA, principal coordinate analysis; LEfSe, linear discriminant analysis effect size.


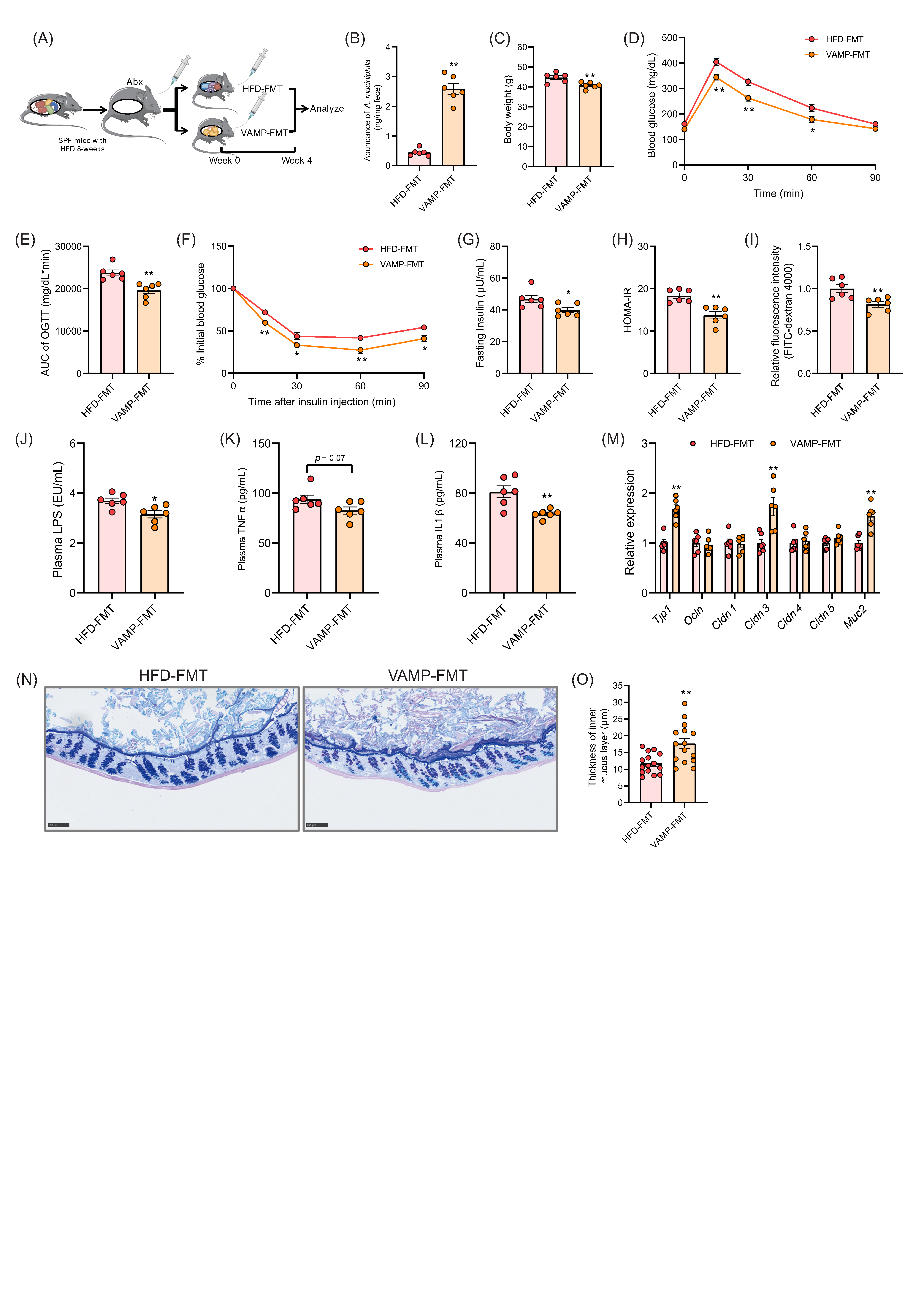


**Figure S5 VAMP treatment improved gut microbiota and glucoregulatory effects can be transfered via FMT.** (A) The experimental scheme for B to O is shown, *n* = 6 mice/group. Mice were fed a HFD for 8 weeks and treated with Abx for 1 week. Then, faecal homogenates from VAMP-treated or untreated HFD-fed mice were orally transferred to Abx-treated recipient mice. (B) *A. muciniphila* abundance in the faeces was assessed by qPCR. (C) The body weights, (D) OGTT curve, (E) AUC of the OGTT, (F) ITT, (G and H) fasting insulin levels and HOMA-IR index of HFD-FMT and VAMP-FMT mice were evaluated. (I) Intestinal permeability was measured by plasma fluorescence intensity after gavage with FITC-dextran 4000. (J) The plasma levels of LPS, (K) TNFα, and (L) IL-1β were determined. (M) The relative expression of *Tjp1*, *Ocln*, *Cldn1*, *Cldn3*, *Cldn4,* and *Cldn5* mRNAs in colonic tissue was evaluated. (N) Carnoy-fixed colonic tissue sections were stained with Alcian blue/periodic acid-Schiff. Scale bars, 100 µm. (O) Blinded colonic mucus layer measurements of Alcian blue-stained sections were performed. All the data are presented as the mean ± SEM. **p* < 0.05 and ***p* < 0.01 versus the HFD-FMT group. HFD, high fat diet; OGTT, oral glucose tolerance test; HOMA-IR, homeostatic model assessment of insulin resistance; TNFα, tumor necrosis factor α; IL-1β, interleukin-1β; LPS, lipopolysaccharide; FMT, fecal microbiota transplantation; AUC, area under the curve.


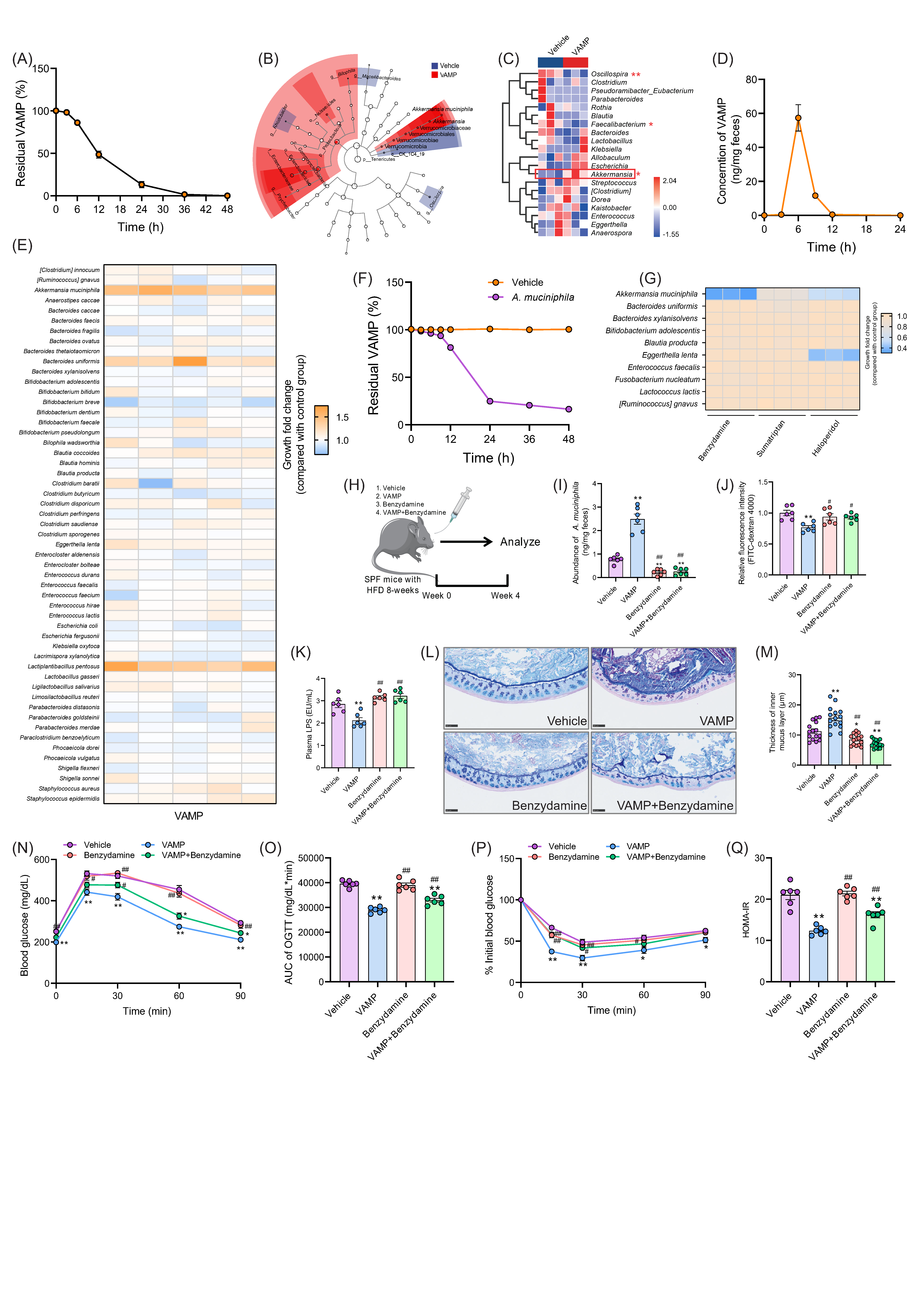


**Figure S6 VAMP improves host glucose metabolism by promoting the expansion of *A. muciniphila.*** (A) The level of remaining VAMP in the medium during incubation with faecal bacteria. (B) Taxonomic cladograms were generated by LEfSe analysis. (C) A heatmap was used to compare the gut microbiota between the vehicle and VAMP groups at the genus level. (D) Mice were gavaged with VAMP (20 mg/kg), and the faeces were collected to measure the level of faecal VAMP. (E) Heatmap showing the effect of VAMP (50 μM) incubation on the growth of gut bacteria at the single-strain culture level. (F) The level of remaining VAMP following incubation with VAMP and *A. muciniphila*. (G) Heatmap showing the effect of different drugs (50 μM) on the growth of *A. muciniphila*. (H) The experimental scheme for I to Q is shown, *n* = 6 mice/group. Mice were fed a HFD for 8 weeks and then treated with PBS (vehicle group), VAMP (50 mg/kg, VAMP group), benzydamine (50 mg/kg, benzydamine group), or VAMP plus benzydamine (50 mg/kg, VAMP+benzydamine group) three times per week for 4 weeks. (I) *A. muciniphila* abundance in the faeces was assessed by qPCR. (J) Intestinal permeability was measured by determining the plasma fluorescence intensity after FITC-dextran 4000 gavage. (K) The plasma LPS level was determined. (L) Carnoy-fixed colonic tissue sections were stained with Alcian blue/periodic acid-Schiff. Scale bars, 100 µm. (M) Blinded colonic mucus layer measurements from Alcian blue-stained sections were performed. (N) The OGTT curve, (O) AUC of the OGTT, (P) ITT curve, and (Q) HOMA-IR index of different groups were evaluated. All the data are presented as the mean ± SEM. **p* < 0.05 and ***p* < 0.01 versus the vehicle group; #*p* < 0.05 and ##*p* < 0.01 versus the VAMP group. LEfSe, linear discriminant analysis effect size; HFD, high fat diet; LPS, lipopolysaccharide; OGTT, oral glucose tolerance test; HOMA-IR, homeostatic model assessment of insulin resistance; ITT, insulin tolerance test.
